# Special Morphological Features at the Interface of the Renal Stem/Progenitor Cell Niche Force to Reinvestigate Transport of Morphogens During Nephron Induction

**DOI:** 10.1089/biores.2015.0039

**Published:** 2016-01-01

**Authors:** Will W. Minuth, Lucia Denk

**Affiliations:** Department of Molecular and Cellular Anatomy, University of Regensburg, Regensburg, Germany.

**Keywords:** cell-to-cell connection, interface, kidney, stem/progenitor cell niche, transport of morphogens, tunneling nanotubes

## Abstract

Formation of a nephron depends on reciprocal signaling of different morphogens between epithelial and mesenchymal cells within the renal stem/progenitor cell niche. Previously, it has been surmised that a close proximity exists between both involved cell types and that morphogens are transported between them by diffusion. However, actual morphological data illustrate that mesenchymal and epithelial stem/progenitor cell bodies are separated by a striking interface. Special fixation of specimens by glutaraldehyde (GA) solution including cupromeronic blue, ruthenium red, or tannic acid for electron microscopy depicts that the interface is not void but filled in extended areas by textured extracellular matrix. Surprisingly, projections of mesenchymal cells cross the interface to contact epithelial cells. At those sites the plasma membranes of a mesenchymal and an epithelial cell are connected via tunneling nanotubes. Regarding detected morphological features in combination with involved morphogens, their transport cannot longer be explained solely by diffusion. Instead, it has to be sorted according to biophysical properties of morphogens and to detected environment. Thus, the new working hypothesis is that morphogens with good solubility such as glial cell line-derived neurotrophic factor (GDNF) or fibroblast growth factors (FGFs) are transported by diffusion. Morphogens with minor solubility such as bone morphogenetic proteins (BMPs) are secreted and stored for delivery on demand in illustrated extracellular matrix. In contrast, morphogens with poor solubility such as Wnts are transported in mesenchymal cell projections along the plasma membrane or via illustrated tunneling nanotubes. However, the presence of an intercellular route between mesenchymal and epithelial stem/progenitor cells by tunneling nanotubes also makes it possible that all morphogens are transported this way.

## Introduction

An increasing incidence of acute and chronic kidney diseases in industrial countries is a serious problem to public health. Although conventional therapies such as hemodialysis and transplantation are available, for some years an alternative therapy for the repair of damaged renal parenchyma by the help of implanted stem/progenitor cells is under development.^1^ To find a reliable source for the regeneration of damaged nephrons and the delivery of supporting molecules including morphogens, various types of stem/progenitor cells were tested.^2,3^ However, the present data show that a real breakthrough for an effective regeneration of diseased renal parenchyma is still not in sight.^4,5^ One of the unsolved problems is the minimal survival of implanted cells.^6^ Another main problem is the hidden cell biological risk, when stem/progenitor cells with uncertain developmental potency are implanted.^7–10^

Against this difficult backdrop it is being considered to apply renal stem/progenitor cells as an original resource for the repair of diseased parenchyma. In this regard a therapeutic activation of quiescent stem/progenitor cells in adult parenchyma^11^ or an implantation of stem/progenitor cells seeded on a renal biomatrix are under current research.^12,13^ Since a couple of years an increasing interest is also directed for gaining information about a therapeutic reactivation of the earlier stem/progenitor cell niche.^14^ In this coherence not only the biological potency of contained cells, the signaling of related morphogens but also the microenvironment of the niche during organ development is of special interest. It is generally hoped that co-implantation of a scaffold simulating such an environment will support survival of cells and in turn will help to push regeneration of diseased renal parenchyma.

During the last few years analysis of the renal niche revealed that here contained stem/progenitor cells are embedded in a more complex environment than it was earlier believed. Special features are a spatial separation of mesenchymal and epithelial stem/progenitor cell bodies, in-between an interface with masked extracellular matrix and finally mesenchymal cell projections establishing a cell-to-cell communication via tunneling nanotubes.^15,16^

Of special importance for the next future is therefore to elaborate more details of illustrated features of the interface. Although the here contained textured extracellular matrix yet can be unmasked by special fixation for electron microscopy, only little information is available about the molecular composition of this complex biomaterial.^17^ Also illustrated cell-to-cell communication via tunneling nanotubes raises new questions on the signaling of morphogens in this unique environment. For example, it is unknown, which sort of morphogens is transported in this special environment by diffusion and which is transported by illustrated intercellular communication. To give a first interpretation about recent morphological findings and the resulting possible routes associated with the transport for morphogens through the interface of the renal stem/progenitor cell niche, the present article is written.

## Orientation of Mesenchymal and Epithelial Cells Within the Niche

**FIG. 1.**
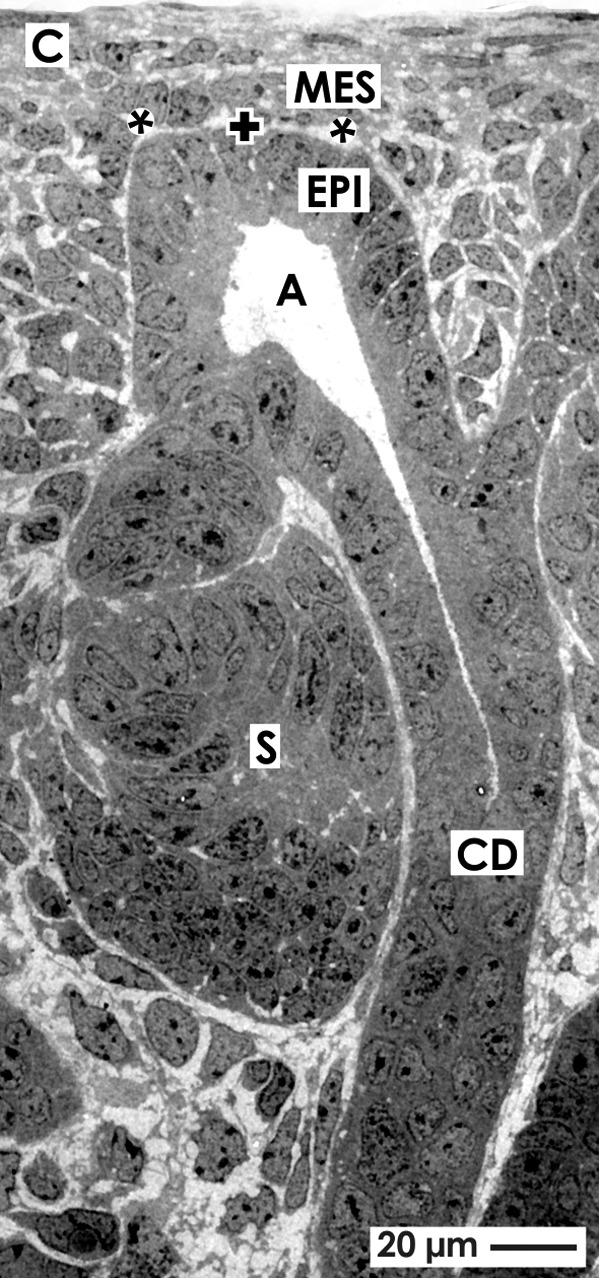
View to the renal stem/progenitor cell niche by transmission electron microscopy. For a reliable perspective the cortex of embryonic parenchyma must be exactly orientated in parallel to a lining collecting duct (CD) and perpendicular to the organ capsule (C). Epithelial (EPI) stem/progenitor cells are seen that are integrated in the tip of a CD ampulla (A), while one to two layers of mesenchymal (MES) stem/progenitor cells surround them. Further mesenchymal cells are separated from epithelial cells by an interface (asterisk). The basal aspect of epithelial stem/progenitor cells at a CD ampulla (A) tip is labeled by a cross (**+**). S marks a developing S-shaped body.

A presupposition for a reliable morphological view to the niche is that fixation of embryonic renal parenchyma has been carefully performed and that histological sections are strictly orientated along the axis of lining collecting duct (CD) tubules (Fig. 1). In this perspective it can be seen that at the inner side of the capsule (C) few layers of special fibroblasts and atypical smooth muscle cells occur.^18^ Beyond them only one to two layers of mesenchymal (MES) stem/progenitor cells are noticed, which are grouped along the basal aspect of an ureteric bud-derived CD ampulla containing epithelial (EPI) stem/progenitor cells. The subpopulation of mesenchymal cells closest to the epithelium of the CD ampulla represents GDNF^+^Six2^+^ cells belonging to the cap mesenchyme (CM).^19,20^ When nephron induction takes place, some of these cells react by performing a mesenchymal-to-epithelial transition (MET) to develop into epithelial cells of the nephron.

Microscopy further illustrates that epithelial and mesenchymal cell bodies are not in direct contact but stand at a distance between 1 and 2 μm (Fig. 1, 2a).^21,22^ This observation is not really new, since years before it was shown by optical microscopy^23–26^ and transmission electron microscopy^27–30^ that an astonishingly wide gap exists around the basal aspect of each CD ampulla. The spatial separation of epithelial and mesenchymal stem/progenitor cells is not restricted to a single species but was also earlier documented in mice, rat, rabbit, and human embryonic renal parenchyma. However, at that time an exact orientation of parenchyma for histological sections was not an issue. It might explain that the separation of mesenchymal and epithelial cells was perceived only casually. Instead, it was typically argued that the gap between both kinds of stem/progenitor cells represents an artifact, which is caused either by inconstant hydraulic force of interstitial fluid or by poor histological preparation.

**FIG. 2.**
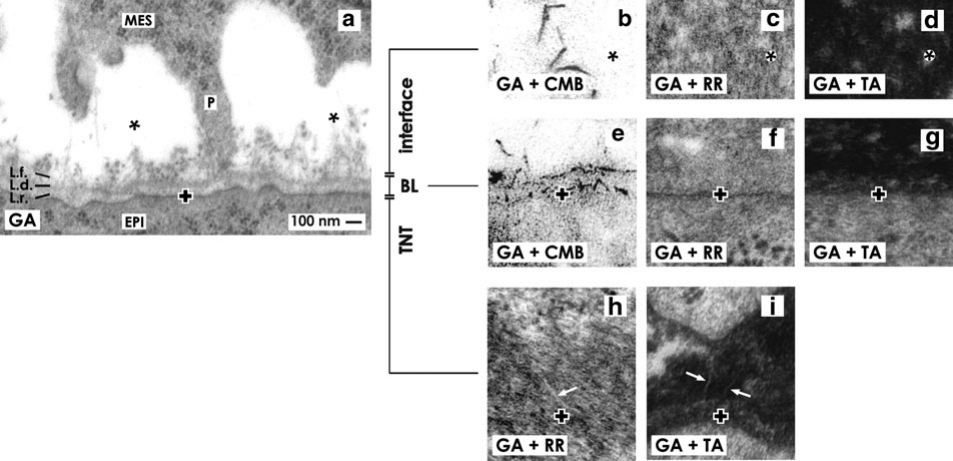
Transmission electron microscopy of the renal stem/progenitor cell niche. **(a)** Fixation of specimens in conventional glutaraldehyde (GA) solution elucidates that an interface (asterisk) is present between mesenchymal (MES) and epithelial (EPI) stem/progenitor cell bodies. Epithelial cells are covered by a basal lamina consisting of a lamina rara (L.r.), lamina densa (L.d.), and lamina fibroreticularis (L.f.). Projections (P) of mesenchymal cells cross the interface to touch the basal lamina of epithelial cells. Within the interface only few extracellular matrix is recognized. In contrast, samples fixed by GA solution including cupromeronic blue (CMB) show that numerous braces of proteoglycans are contained **(b)** on the surface of mesenchymal cell projections and **(e)** in the basal lamina. Specimens fixed by GA solution including **(c, f)** ruthenium red (RR) or (**d, g**) tannic acid (TA) illuminate earlier nonvisible extracellular matrix within the interface and on the basal lamina. **(h, i)** Tunneling nanotubes (arrow) establish a cell-to-cell connection between mesenchymal and epithelial cells. The basal plasma membrane of epithelial stem/progenitor cells is labeled by a cross (**+**).

## The Interface Between Mesenchymal and Epithelial Cells

To reinvestigate more precisely the obvious separation of mesenchymal and epithelial cell bodies within the renal stem/progenitor cell niche, transmission electron microscopy with correctly orientated sections was performed. As everybody knows, for some reasons traditional glutaraldehyde (GA) solution has prevailed as the golden standard for histological fixation. When this technique is applied for embryonic renal parenchyma, transmission electron microscopy demonstrates that a polarized monolayer of epithelial cells is enclosed in the tip of a CD ampulla. Its basal aspect is covered by a basal lamina consisting of a lamina rara (L.r.), lamina densa (L.d.), and lamina fibroreticularis (L.f.) (Fig. 2a). Already years ago it was shown that in the lamina fibroreticularis a special fibrillar mesh-work is contained.^31^ In close neighborhood a cell body is seen belonging to the surrounding CM. There it is also recognized that the body of a mesenchymal cell does not touch but has a distance between 1 and 2 μm to the basal lamina of an opposite epithelial cell. It is obvious that in-between them a bright but unobtrusive looking interface is visible (Fig. 2a; asterisk).

Specimens fixed in traditional GA solution additionally reveal that projections (also called cytonemes or filopodia) of a mesenchymal cell cross the interface to contact the basal lamina of an epithelial cell. Surprisingly, only few and barely visible microfibers of extracellular matrix are seen that originate at the lamina fibroreticularis, cross the interface, and contact a mesenchymal cell.

## Unmasking of Textured Extracellular Matrix Within the Interface

Although fixation of specimens by traditional GA solution gives the impression of a void interface, an always constant distance between mesenchymal and epithelial cell bodies is observed (Figs. 1 and 2a). For that reason it was assumed that the interface cannot be incidentally caused by hydraulic force of interstitial fluid. Instead, it has been suggested that it is based on masked extracellular matrix as it was earlier described.^32^ To obtain more information about suspected structural details, alternative fixation by GA solution including cupromeronic blue, ruthenium red, or tannic acid was performed.^17^

Fixation of specimens in GA solution including cupromeronic blue illustrates numerous braces of proteoglycans (Fig. 2b, e). One sort of them is detected on the surface of mesenchymal cell projections (Fig. 2b). At the end of a projection braces of proteoglycans form a sleeve to mount it on the lamina fibroreticularis. Another sort of them forms chains along the basal plasma membrane and the lamina fibroreticularis (Fig. 2e).

Specimens fixed in GA solution including ruthenium red unmasks extended textural extracellular matrix within the interface. This result depicts that the interface is not void as earlier believed but filled out to a high degree by a scaffold consisting of illustrated extracellular matrix (Fig. 2c). Further it is recognized that fuzzy extracellular matrix covers the surface of crossing mesenchymal cell projections. Label by ruthenium red also presents that in the basal lamina of epithelial cells an earlier not visible dense band of extracellular matrix is contained. However, applying this label it is not anymore possible to differentiate between the lamina rara, lamina densa, and lamina fibroreticularis (Fig. 2f).

Fixation of specimens in GA solution including tannic acid reveals that in extended areas of the interface a dense but punctual label of textured extracellular matrix is contained (Fig. 2d). Further a coat of fuzzy extracellular matrix is detected on the surface of crossing mesenchymal cell projections. On the basal lamina of epithelial cells tannic acid label illustrates a broad band of extracellular matrix (Fig. 2g). Also in this staining profile a discrimination between the lamina rara, lamina densa, and lamina fibroreticularis is not anymore possible.

Thus, alternative fixation of specimens in GA solution including cupromeronic blue (Fig. 2b, e), ruthenium red (Fig. 2c, f), or tannic acid (Fig. 2d, g) unmasks textured extracellular matrix that was earlier not visible but is definitively part in extended areas of the interface.^22^ It is obvious that this matrix forms a filigree scaffold that in turn causes the spatial separation of mesenchymal and epithelial stem/progenitor cell bodies. Surprisingly, also mesenchymal cell projections crossing the interface are integrated in this scaffold.^15,16^

## Cell-to-Cell Contacts Between Mesenchymal and Epithelial Cells

Although the bodies of mesenchymal and epithelial stem/progenitor cells are separated by a striking interface within the niche, in the electron microscope can be seen that projections of mesenchymal cells cross it to contact the basal lamina of epithelial cells (Fig. 2a). Moreover, when the section plane shows a projection near the basal lamina, it is recognized that it does not dangle but penetrates the basal lamina. At this site the ending of a projection is surrounded by extracellular matrix that forms a special sleeve to ensure the contact.^15,16^

An important question is, which sort of molecules establishes the illustrated cell contact. Although little information is available, it appears most probable that at the end of a mesenchymal cell projection integrin α8β1 is localized, which binds to nephronectin as its receptor within the basal lamina of an epithelial cell as it was described earlier.^33–35^ Also the microtubule-dependent motor protein kinesin KIF26B was shown in comparable projections possibly involved in regulating attraction of cells, signal transduction, or developmental patterning.^36,37^ However, recently performed immunohistochemical experiments in our laboratory with antibodies reacting against related proteins did not show clear evidence, so that this issue yet cannot be confirmed.

Surprisingly, decades ago comparable cell contacts were demonstrated on embryonic mouse kidney but were not further cited in literature.^38^ In contrast to our investigation at that time microscopic analysis was not performed on neonatal kidneys but at the stage of organ formation, when the ureteric bud has branched only once. First of all, it was observed that mesenchymal cells are separated from the ureteric bud by an “interspace.” In addition, cytoplasmic processes were documented that cross the ample interspace (see in this paper related figure 2).^38^ Finally, high enlargement in electron microscopy elucidated that mesenchymal and epithelial cells are connected via cell processes (see in this article related figure 8).^38^ Thus, these earlier observations support our present data and show that intercellular contacts exist not only during the initial phase of organ formation but also in neonatal kidney.^15,16^

## Intercellular Communication Via Tunneling Nanotubes

Transmission electron microscopy further reveals that projections of mesenchymal cells cross the interface, penetrate the basal lamina, and establish a contact with epithelial cells (Fig. 2a). High enlargement further illustrates that the end of a mesenchymal cell projection and the basal plasma membrane of an epithelial cell are approaching but surprisingly do not simply fuse. Instead, they stay at an average distance of 167 nm.^16^

By the first view, this result speaks only for a mechanical contact between the end of a mesenchymal cell projection and an epithelial cell. However, at a second glance one can see that in the end of a projection, in the approaching zone and in the basal plasma membrane of an epithelial cell tunneling nanotubes are present (Fig. 2h, i).^15,16^ This unexpected finding illustrates that an intercellular route via tunneling nanotubes exists that is principally suited for intercellular communication and transport of a multitude of molecules including even cell organelles.^39^ Thus, renal stem/progenitor cells within the niche cannot be longer defined as an assembly of more or less isolated hermits, but now they must be regarded as a network of unexpectedly communicating cells.

## Cell-to-Cell Mediated Signaling

Molecular signaling and communication between embryonic renal cells had long been an issue. Already in the 1950s Clifford Grobstein investigated the exchange of morphogenetic information. At that time morphogens as individual molecules were envisaged but could not be really verified. To obtain nonetheless detailed information, transfilter culture experiments with mouse metanephrogenic mesenchyme were performed.^40^ In those experiments isolated mesenchyme was placed on the one side, while spinal cord—not ureteric bud—as an effective inducer tissue was placed on the other side of a filter simulating a permeable interface.^41,42^ This special culture set up revealed that success of induction recorded in form of developing tubules depends on several parameters such as thickness, porosity, and pore size of the inserted filter. It was further shown that a pore size below 0.1 μm prevents extension of cell-to-cell contacts and in turn blocks development of tubules.^43^ It was also demonstrated that a transfilter contact between the interacting cells is established within 1 h provided that cytoplasmic processes emerge through the interposed filter. Then an unexpected long lag period of 16–24 h is needed for completion of induction.

In the meantime morphogens were identified. Performing a second generation of transfilter culture experiments, Wnt4-expressing NIH3T3 cells were used to induce mesenchyme instead of spinal cord. It was demonstrated that separating filters with pore sizes of 0.1 μm and above supported induction of tubule formation, whereas pores of 0.05 μm abolished it. Finally, soluble molecules in form of a supernatant from Wnt4-expressing cells were not able to induce formation of tubules.^44^ Looking over all, these earlier results point out that the transport of a morphogen during induction of a nephron is not as easy as it looks, cannot be explained alone by diffusion of a soluble morphogen, and is certainly also based on contacting cell projections.^45^

## Signaling of Morphogens Within the Niche

Stem/progenitor cells stay within the niche from the beginning of organ formation up to the neonatal period of the kidney.^46^ During this phase contained mesenchymal and epithelial stem/progenitor cells are exposed to a manifold signaling of morphogens to maintain on the one hand self-renewal and on the other hand differentiation including formation of new nephrons.^47,48^ One of significant morphogen actions is performed by Mdm2 triggering survival, proliferation, and competence. Its intact signaling is recognized by expression of typical progenitor markers such as Amphiphysin, Cited1, Sall1, and Pax 2.^49^ Another main task of signaling is to control the synthesis, secretion, and transport of morphogens initiating induction and the subsequent development of the nephron. In this special group heterogeneously composed molecules are included known also as morphogenetic proteins, peptides, and growth factors.^50^

Induction of a nephron actually starts, when the branching of an ureteric bud-derived tip of a CD ampulla is terminating.^51^ For a short period of time a single layer of GDNF^+^Six2^+^ cells of the CM is yet exposed to the basal side of epithelial cells for a reciprocal signaling of a series of morphogens. Molecules specifically involved in this process are Glial cell line-Derived Neurotrophic Factor (GDNF), Bone Morphogenetic Proteins (BMP4 and BMP7), WNT family members (Wnt4, Wnt5a, and Wnt9b), and Fibroblast Growth Factor (FGF8).^52–57^ For a productive transmission of these signals also related receptors such as Fgfr1, Fgfr2, Gfra1, Notch 2, Ret tyrosine kinase receptor, and transcription factors such as BRN1, FoxC2, LIMI, Osr1, Sall1, Pax2, and Wt1 are needed.^50^

As a result of this pleiotropic signaling during nephron induction few GDNF^+^Six2^+^ cells separate and shift to the lateral side of a related CD ampulla. Here they perform a MET and develop into a pretubular aggregate and then in a renal vesicle as the first visible sign of a developing nephron.^58^

## Diffusion of Morphogens Versus Controlled Transport

It is well known that induction of a nephron depends on an intact signaling of various morphogens between mesenchymal and epithelial stem/progenitor cells.^50,51^ Although an effective respectively missing signaling was intensively investigated by numerous experiments with transgenic animals, surprisingly little information was given about biophysical properties and the concrete transport of mentioned morphogens.^59,60^

**FIG. 3.**
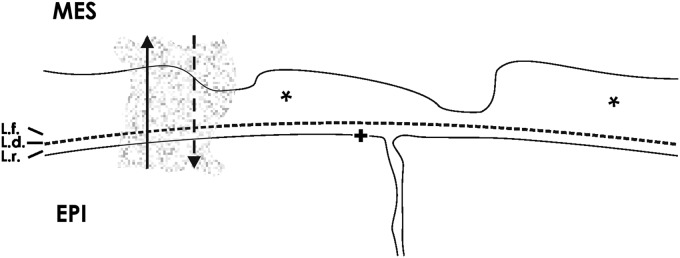
Schematic illustration informs about the exchange of morphogens within the renal stem/progenitor cell niche in an earlier view. For nephron induction it was assumed that mesenchymal (MES) and epithelial (EPI) stem/progenitor cells have an intimate contact. Under such conditions all morphogens are transported by diffusion (arrows) through the interstitial space (asterisk). The basal lamina consisting of a lamina rara (L.r.), lamina densa (L.d.), and lamina fibroreticularis (L.f.) covers the basal aspect of epithelial cells. The basal plasma membrane of epithelial cells is marked by a cross (**+**).

For successful signaling it was previously supposed that mesenchymal and epithelial stem/progenitor cells have an intimate contact, the space between them is filled by negligible interstitial fluid, and all of the involved morphogens have more or less the same biophysical properties in saline (Fig. 3). Consequently, it was presupposed that all morphogens are transported by diffusion. Under such ideal conditions the route for diffusing morphogens is minimal and loss by dilution in interstitial fluid is unattended and small. In turn, a sharp gradient can be built up, so that an effective concentration of a morphogen will reach its receptor as it was earlier communicated.^61^

However, earlier^38^ and actual^15,16^ literature including present morphological data (Fig. 2) contradict the general assumption that all involved morphogens are transported by diffusion between mesenchymal and epithelial cells. A clear reason from the biophysical point of view is that each kind of morphogen has individual molecular properties resulting in a good, minor, or poor solubility in saline. Clear morphological reasons are the spatial separation of mesenchymal and epithelial cell bodies, in-between a striking interface filled to a high degree with textured extracellular matrix including an intact basal lamina covering epithelial cells (Fig. 2b–g). Further, the label by cupromeronic blue (Fig. 2b, e) indicates that in the interface proteoglycans are contained that can strongly influence transport of morphogens by binding.^62^ Finally, the presence of an intercellular communication between mesenchymal cell projections and epithelial cells via tunneling nanotubes point out that an earlier not considered route for a controlled transport of molecules exists (Fig. 2h, i).^63^

Thus, different biophysical properties of involved morphogens and detected morphological details within the renal stem/progenitor cell niche have to be brought on a common denominator. Consequently, a first attempt is made to sort involved morphogens according to good,^20,52,64^ minor,^65,66^ and poor^67–69^ solubility in saline. Following this consideration it is possible to allocate the transport of morphogens to the morphological findings presented here (Table 1 and Fig. 2). Since for the renal stem/progenitor cell niche such a concept did not exist earlier, the following considerations were made according to new morphological findings^15,16^ and to cell biological data raised in other developmental systems.^70^

**Table 1.** **Morphogens Involved in Signaling During Nephron Induction Were Sorted According to Biophysical Features**

Morphogen	Solubility of morphogen in saline	Diffusion	Binding on extracellular matrix	Cell projection TNT	Reference
GDNF	++++	+			Michos et al.,^52^ Combes et al.^20^
FGF8	++++	+	+		Abuharbeid et al.^64^
BMP4	+		+	+	Swencki-Underwood et al.,^65^ Pohl et al.^66^
BMP7	+		+	+	Swencki-Underwood et al.,^65^ Pohl et al.^66^
Wnt4	(0)		+	+	Gross and Boutros^68^
Wnt5a	(0)		+	+	Gross and Boutros^68^
Wnt9b	(0)		+	+	Gross and Boutros^68^
Shh	(0)		+	+	Bandari et al.,^69^ Creanga et al.^67^

They exhibit either a good (++++), minor (+) or poor (0) solubility in saline. Then detected morphological features in the interface of the niche were allocated with indicated biophysical properties. It is concluded that morphogens with good solubility are transported by diffusion, while morphogens with minor solubility bind after secretion in extracellular matrix and morphogens with poor solubility are transported in cell projections and via TNTs.

TNT, tunneling nanotube.

## Morphogen Transport by Diffusion

Label by cupromeronic blue (Fig. 2b, e), ruthenium red (Fig. 2c, f), and tannic acid (Fig. 2d, g) exhibits that extended areas of the interface are filled by a scaffold of textured extracellular matrix. Hence, the complementary but much minor space does not exhibit any label, appears to contain only interstitial fluid, and is consequently best suited for diffusion of molecules. Due to its biophysical properties a candidate for a transported morphogen is GDNF (Table 1). It consists of 134 amino acids, is secreted as a glycoprotein, and is therefore readily soluble in saline respectively interstitial fluid.^71^ Surprisingly, only GDNF synthesized by mesenchymal cells was up to date defined as such a long-distance diffusible morphogen that binds on Ret tyrosine kinase receptor and a co-receptor GFRα1 located at the tip of a CD ampulla.^20,72^

## Morphogen Deposition and Transport in Extracellular Matrix

Label of cupromeronic blue (Fig. 2b) on mesenchymal cell projections indicates presence of syndecans and/or glypicans, while ruthenium red (Fig. 2c, f) or tannic acid (Fig. 2d, g) label within the interface speaks for the presence of perlecans and other proteins of extracellular matrix.^73^ From these proteoglycans it is known that they are central modulators of kidney development by interacting with morphogens such as GDNF, members of the FGF and TGFβ superfamilies, EGF receptor ligands, and HGF (Table 1).^74–77^ The conception is that a binding of these molecules on proteoglycans acts as a “morphogenetic switch” influencing either inhibitors or facilitators as the fine tuning of a morphogen gradient. The importance of extracellular matrix is further recognized by the fact that environment lacking heparan sulfate proteoglycans does not support formation of an effective Wnt gradient and in turn prevents further development.^73^

## Morphogen Transport Via Cell Projections

Wnt4, Wnt5a, and Wnt9b are essential morphogens for renewal and differentiation of nephron progenitors, CD ampulla branching, and nephron induction.^50,59^ Further Wnts contain post-translational modifications in the form of a saturated palmitic acid and an unsaturated palmitoleic acid.^68^ Due to these specific modifications on the molecular structure they have a poor solubility in interstitial fluid (Table 1). For that reason it is also most likely that they are not widely sprayed into the interstitial space by diffusion but secreted in the vicinity of illustrated cell projections (Fig. 2a). In the case they reach the plasma membrane of a target cell, it is further imaginable that they bind to a cargo that transports Wnts.^78,79^ In analogy to mammalian kidney, Drosophila Tkv-GFP receptor puncta in cell projections were detected that are able to move here either in an anterograde or retrograde direction.^80^

Sonic hedgehog (Shh) is a morphogen that controls renal patterning.^81^ This special molecule is not secreted into the interstitial space and transported by diffusion but is produced in form of a particle. Surprisingly, the particles remain associated during transport with the cell surface on long cytoplasmic extensions (projections) that can span over several cell diameters.^82,83^

Besides lipophilic Wnts and plasma membrane-associated Shh, the group of BMPs also belongs to morphogens with minor solubility in interstitial fluid.^20^ For that reason the transport of a BMP through the interface by diffusion is unlikely (Table 1). Instead, transport at the contact between a mesenchymal cell projection and an epithelial cell appears to be more probable. At this site BMP can bind at the plasma membrane^84^ and transported with its receptor via microtubules as it was demonstrated for Drosophila Tkv.^85^

## Morphogen Transport in Tunneling Nanotubes

Actual morphological data show that projections of mesenchymal cells cross the interface and the adjacent basal lamina to establish a contact with epithelia cells at the tip of a CD ampulla (Fig. 2a). At the end of a projection and the basal plasma membrane of an epithelial cell tunneling nanotubes are integrated establishing here an intercellular communication and transport system (Fig. 2h, i).^16^

Generally, transport in tunneling nanotubes includes an intercellular transfer of organelles, membrane compounds, and cytoplasmic molecules.^86–91^ Although it was not proven, it appears most probable that also morphogens maintaining stemness and triggering nephron induction are transported here. However, up to date those functions in combination with tunneling nanotubes were not described for the embryonic kidney, but were investigated by *in vitro* experiments with renal cells but in different experimental coherence.^39,92^ For that reason more morphological details about illustrated tunneling nanotubes, extension at the contact site, molecular construction, colocalization with other proteins and individual transport features within the renal niche wait to be generated.^93^

## Diffusion Versus Directed Transport of Morphogens

The transport of morphogens within the renal stem/progenitor cell niche was in the past more simplified described than it really seems to be (Fig. 3). Recently detected morphological details in the renal stem/progenitor cell niche demonstrate a spatial separation of mesenchymal and epithelial cell bodies, in-between a structured interface filled to a high degree with textured extracellular matrix, crossing projections of mesenchymal cells, cell-to-cell contacts, and intercellular communication via tunneling nanotubes (Fig. 2).^15,16^ These morphological details in sum make an exclusive transport of all morphogens by diffusion unlikely. Consequently, the proposal is that transport of morphogens is classified according to illustrated morphological details (Fig. 2) and according to biophysical properties of involved morphogens (Table 1). By the first view such a concept appears to be questionable for the renal stem/progenitor cell niche but was earlier outlined for other developmental systems such as Drosophila or Zebrafish.^94,95^

**FIG. 4.**
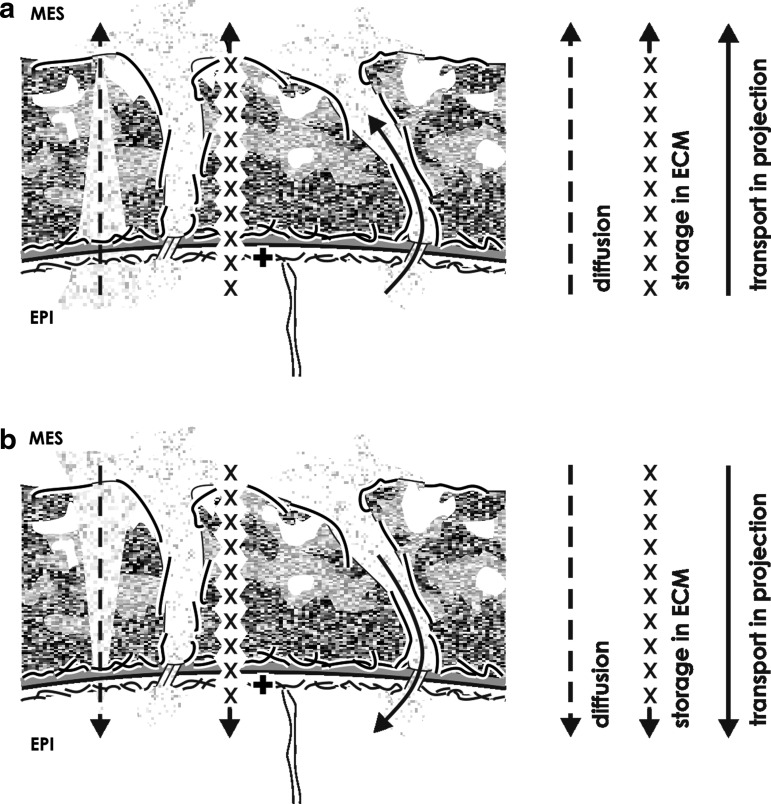
Schematic illustration informs about the exchange of morphogens within the renal stem/progenitor cell niche in an actual view. Detected morphological features show that mesenchymal and epithelial cells are separated by an interface including a basal lamina and abundant extracellular matrix. Further mesenchymal cell projections cross the interface to establish a cell-to-cell communication with epithelial cells. On that special situation it is speculated that only one part of morphogens is transported by diffusion (dashed arrow) from **(a)** an epithelial to a mesenchymal cell or vice versa from **(b)** a mesenchymal to an epithelial cell. The second part of morphogens is secreted and then bound in extracellular matrix (xxx arrow). Here it is decided upon their free accessibility to the target cell or further binding, modification, storage, and delivery on demand. The third part of morphogens is transported by cell projections and tunneling nanotubes (solid arrow) from an epithelial to a mesenchymal cell or vice versa from a mesenchymal to an epithelial cell. The basal aspect of epithelial cells is marked by a cross (**+**).

Based on presented actual morphological data, for the renal stem/progenitor cell niche it is yet assumed that morphogens such as GDNF or FGF8 with a rather good solubility are transported by passive diffusion (Fig. 4 and Table 1). For morphogens such as BMP4 or BMP7 it is suggested that they are transported by restricted diffusion so that they interact after secretion with extracellular matrix detected in the interface. Here, it is decided upon their free accessibility to the target cell or whether they are bound, modified, stored and delivered on special demand. For morphogens such as Wnt4, Wnt5a, Wnt9b, or Shh it is proposed that they are bound in extracellular matrix or transported in illustrated cell projections (Fig. 4 and Table 1). This passage transport of morphogens is thinkable as well on the plasma membrane of a cell projection via tunneling nanotubes in its interior.^96–98^ Finally, regarding mesenchymal cell projections including intercellular communication with epithelial cells via tunneling nanotubes, it is also imaginable that all involved morphogens and independently from their biophysical properties are comfortably transported via tunneling nanotubes.^99^

Theoretically and independent from mentioned routes, transport of morphogens may also occur by vesicles such as exosomes (40–100 nm) or microvesicles (100–1000 nm).^100,101^ By this mechanism as well mRNA or microRNA as an synthesized morphogen molecule can be shuttled.^102,103^ However, up to date no information is available, whether vesicles are involved in the transport of morphogens within the renal stem/progenitor cell niche.

## Translational Aspects of Research

The detected microarchitecture within the renal stem/progenitor cell niche and the special contact between mesenchymal and epithelial stem/progenitor cells shed new light on their life within a special environment and their sociality communicated via cell-to-cell contacts. As a consequence, it is now time to thoroughly investigate the individual transport of morphogens and the intercellular communication between involved cells by actual cell biological techniques. Taking further into account the unique extracellular microenvironment within the niche, it is worthwhile to think about its biomedical simulation and then about a practical application for the repair of diseased renal parenchyma. It is imaginable to develop, for example, a biodegradable but smart scaffold with seeded stem/progenitor cells and/or integrated morphogens that is able to ensure niche environment for the initial time after an implantation has been performed. Regarding further the avascular environment of the niche, there is a likelihood that only such an introduced scaffold will support survival of stem/progenitor cells in the harmful environment within diseased renal parenchyma. Moreover, beside restoration of renal functions for elderly patients, intact generation of parenchyma has a special meaning for preterm infants. In those cases the nephrogenic potential has to be stabilized by innovative biomedicine to prevent developmental alterations of the kidneys and consequently chronic kidney disease later in life.^104^

## Conclusions

Previously it was assumed that mesenchymal and epithelial cells in the renal stem/progenitor cell niche have an intimate contact and that the reciprocal transport of morphogens during induction of a nephron is based exclusively on diffusion. However, recent morphological findings illustrate that mesenchymal and epithelial cell bodies are separated by a striking interface consisting of textural extracellular matrix. Further on, projections of mesenchymal cells cross the interface to establish an intercellular communication with epithelial cells via tunneling nanotubes. Regarding the heterogeneously composed group of involved morphogens in combination with the special microenvironment in the interface and the presence of tunneling nanotubes, an exchange of morphogens alone by diffusion seems highly unlikely. Instead, due to flexibility of mesenchymal cell projections including tunneling nanotubes, it is probable that most of morphogens are transported this path at the right time, punctual site, and dosed amount. Whether microvesicles are involved in the transport of morphogens within the renal stem/progenitor cell niche has to be explored.

## Abbreviations Used

BMP: bone morphogenetic proteins
CD: collecting duct
CM: cap mesenchyme
FGF: fibroblast growth factor
GA: glutaraldehyde
GDNF: glial cell line-derived neurotrophic factor
MET: mesenchymal-to-epithelial transition
